# Can supplementation of tryptophan in parenteral nutrition increase melatonin and alleviate inflammatory response?

**DOI:** 10.1590/1806-9282.20230826

**Published:** 2024-04-22

**Authors:** Necdet Fatih Yaşar, Bartu Badak, Mustafa Salış, Fatih Kar, Setenay Öner

**Affiliations:** 1 Osmangazi University, Faculty of Medicine, Department of General Surgery – Eskişehir, Turkey.; 2 Eskişehir City Hospital, Department of General Surgery – Eskişehir, Turkey.; 3 Kütahya Health Sciences University, Faculty of Engineering and Natural Sciences, Department of Biochemistry – Kütahya, Turkey.; 4 Osmangazi University, Faculty of Medicine, Department of Biostatistics – Eskişehir, Turkey.

**Keywords:** Melatonin, Tryptophan, Inflammatory response, Surgery, Nutrition, Parenteral nutrition

## Abstract

**OBJECTIVE:**

Endogenous melatonin is produced from tryptophan which is an essential amino acid. Besides its role in the regulation of sleep patterns, melatonin has anti-inflammatory effects. In this case-control study, we aimed to compare tryptophan and melatonin levels and their relationship with the inflammatory response, specifically serum interleukin-1, interleukin-6, and c-reactive protein levels following major abdominal surgery in patients with food restriction and who receive parenteral nutritional therapy.

**METHODS:**

We enrolled 40 patients between the ages of 18 and 65 years in the study. We collected blood and urine samples 48 h before the operation and on postoperative days 1, 3, and 5.

**RESULTS AND CONCLUSION:**

The tryptophan levels in the experimental group were higher than in the control group but failed to reach any statistical difference. Melatonin levels were increased in both groups following the surgery compared with preoperative levels. The increase in the experimental group was statistically different 3 days after the surgery. The difference in the level of interleukin-1 between the control and the experimental groups was greatest on postoperative day 3. On postoperative day 3, the interleukin-6 level in the treatment group was slightly higher than in the control group. We did not find any difference in the levels of c-reactive protein between the groups. As a result, the levels of tryptophan and melatonin were increased in the parenteral nutrition group, irrespective of the postoperative inflammatory response.

## INTRODUCTION

Endogenous melatonin is produced from tryptophan which is an essential amino acid^1^. Besides its role in the sleep patterns, melatonin has anti-inflammatory effects. Studies have shown that melatonin may counteract proinflammatory response^2–5^. Thus, the interaction between inflammation and melatonin secretion seems to be essential for hemostasis after surgery. In this regard, our previous study has shown that improving sleep quality may ameliorate inflammatory response after major abdominal surgery by increasing melatonin secretion^6^.

It has been shown that acute tryptophan depletion in healthy volunteers decreased plasma tryptophan levels as well as melatonin levels^7,8^. On the contrary, we have observed that postoperative melatonin levels increase in our previous study regardless of sleep quality^6^. However, there is no study about the effects of postoperative food restriction on tryptophan levels and melatonin secretion. In this case-control study, we compared tryptophan and melatonin levels and their possible relationship with the inflammatory response following abdominal operations in patients with food restriction and in patients who receive parenteral nutritional therapy.

## METHODS

This study has been approved by the Ethics Committee of Eskisehir Osmangazi University and conducted in accordance with the principles of the Declaration of Helsinki (No. E-25403353-050.99-93363). We enrolled 40 patients between the ages of 18 and 65 years who underwent abdominal operations in the study. Informed consents were taken from all patients. Patients with inflammatory diseases and hormone-related conditions, such as medication or neoplasms, and patients with malnourishment were excluded. Patients were divided into two groups, the dietary restriction group and the parenteral nutritional therapy group [Oliclinomel N4-550E, (Baxter, Turkey) which had 0.040 g/L of tryptophan], depending on the anticipation if the patient would be able to start eating after 5 days postoperatively.

Preoperative blood and urine samples were collected 48 h before the operation. The demographics of the patients were recorded. Postoperative samples were collected on days 1, 3, and 5. We monitored melatonin production by the determination of urine 6-sulfatoxymelatonin (aMT6s). A 24-h urine sample was collected. Besides urine aMT6, serum tryptophan levels were measured. We monitored inflammatory response by measuring parameters, including interleukin-1 (IL-1), interleukin-6 (IL-6), and c-reactive protein (CRP).

We assayed the urinary aMT6 levels using a commercial enzyme-linked immunosorbent assay (ELISA) kit [IBL International GmbH ELISA kit (Hamburg, Germany)]. We analyzed serum levels of IL-1, IL-6, and tryptophan using the Bioassay Technology Laboratory ELISA kits (Zhejiang, China) and CRP levels using an automated analytical system (SiemensDimension Vista^Ò^ 1500, Siemens Healthcare Diagnostics, Tarrytown, NY, USA).

### Statistics

As the Shapiro-Wilk test showed that the variables were not distributed normally, we compared the experimental and control groups using the Mann-Whitney U test. We evaluated the difference between preoperative, postoperative days (PODs) 1, 3, and 5 variables in both groups using the Tukey's HSD test.

## RESULTS

There was no difference between the control and the experimental groups concerning demographics.

**Figure 1:**
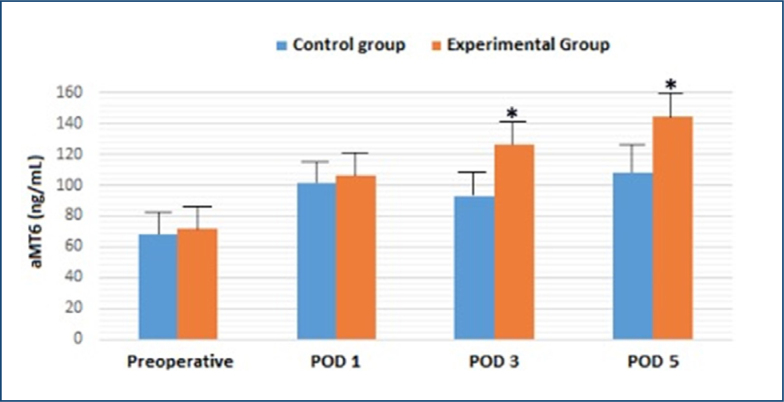
The effects of parenteral nutrition on plasma 6-sulfatoxymelatonin levels after major abdominal surgery. *p<0.05 versus experimental group.

There was no difference between the preoperative and postoperative plasma tryptophan levels in the control group. The tryptophan levels in the experimental group were increased beginning on the third day after the surgery and reached the highest level on POD 5 but failed to reach any statistical difference (Table 1). Melatonin levels were higher in both the control and experimental groups following the surgery compared with preoperative levels, but the increase in the experimental group reached a statistical difference on POD 3 and the peak level on POD 5. Similar to the tryptophan levels, POD 3 and POD 5 melatonin levels were greater in the experimental group than in the control group (Table 1 and Figure 1).

**Table 1:** Basic patient demographics and preoperative and postoperative plasma tryptophan, 6-sulfatoxymelatonin, interleukin-1, interleukin-6, and c-reactive protein levels.

		Control group Median (25–75%)	Experimental group Median (25–75%)	p
Age Gender (number of males)		61.00 (51.50–64.50) 12	61.00 (55.00–64.00) 13	0.860
**Preoperative**				
	Tryptophan (μg/mL)	24.95 (22.14–27.18)	25.70 (20.94–27.34)	0.957
	aMT6 (μg/day)	68.03 (45.03–91.42)	71.64 (59.72–117.21)	0.500
	IL-1 (pg/mL)	34.61 (29.56–68.61)	31.99 (29.08–35.93)	0.062
	IL-6 (pg/mL)	43.08 (30.61–55.79)	41.57 (31.32–53.53)	0.482
	CRP (mg/L)	2.70 (2.25–4.15)	2.25 (1.00–3.45)	0.285
**POD 1**				
	Tryptophan (μg/mL)	24.95 (23.63–28.13)	25.39 (20.50–45.75)	0.818
	aMT6 (μg/day)	101.03 (71.17–139.18)	106.12 (78.47–138.87)	0.844
	IL-1 (pg/mL)	39.99 (30.60–64.51)	36.06 (30.32–40.44)	0.168
	IL-6 (pg/mL)	63.84 (54.20–71.22)	65.44 (50.62–112.84)	0.490
	CRP (mg/L)	86.30 (41.35–119.65)	73.25 (54.30–124.50)	0.957
**POD 3**				
	Tryptophan (μg/mL)	24.75 (24.89–31.97)	28.77 (23.30–57.64)	0.665
	aMT6 (μg/day)	93.27 (65.93–128.37)	126.46 (104.13–189.94)	**0.046**
	IL-1 (pg/mL)	45.65 (35.28–72.89)	32.87 (29.14–40.71)	**0.006**
	IL-6 (pg/mL)	67.54 (58.23–68.95)	75.80 (58.03–100.88)	0.525
	CRP (mg/L)	167.25 (69.50–207.65)	144.50 (107.15–214.80)	0.892
**POD 5**				
	Tryptophan (μg/mL)	23.89 (23.53–32.47)	29.27 (23.34–53.52)	0.285
	aMT6 (μg/day)	108.51 (67.46–127.49)	144.32 (94.48–179.29)	**0.038**
	IL-1 (pg/mL)	36.60 (34.40–40.42)	35.08 (28.54–40.68)	0.185
	IL-6 (pg/mL)	65.04 (61.46–79.73)	64.64 (57.18–110.04)	0.957
	CRP (mg/L)	67.05 (40.10–109.05)	60.30 (35.45–93.95)	0.534

Statistically significant values are indicated in bold.

**Figure 2:**
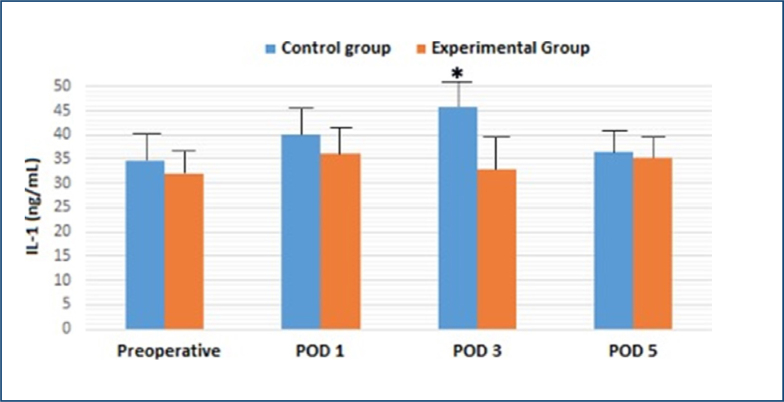
The effects of parenteral nutrition on plasma interleukin-1 levels after major abdominal surgery. *p<0.05 versus experimental group.

The level of IL-1 was increased in both groups on POD 1, and the difference between the control and the experimental group was greatest on POD 3. However, the difference was diminished on POD 5 (Table 1 and Figure 2). On the contrary, the level of IL-6 on POD 3 in the treatment group was slightly greater than in the control group (Table 1).

The levels of CRP were less in the treatment group, and the difference was greatest on POD 3 but statistically not different (Table 1).

## DISCUSSION

In this study, we have observed that the levels of tryptophan and melatonin were increased in the experimental group irrespective of the inflammatory response.

Previous studies by Zimmermann et al., have shown that acute tryptophan depletion reduces plasma tryptophan and melatonin levels in healthy subjects at night^7^. They also showed that urinary 6-SM can be used as a valid and reliable indicator of melatonin production^8^. On the contrary, Ploder et al., did not observe any significant changes in the tryptophan concentrations but increased kynurenine concentrations after tryptophan depletion in trauma patients^9^. Similarly, our results have shown no difference between the preoperative and postoperative plasma tryptophan levels in the control group. However, the tryptophan levels in the experimental group were increased and reached the highest level on POD 5, but the difference was not statistically important. Melatonin levels were higher in both the control and experimental groups, which was consistent with the findings in our previous study in which we investigated how sleep quality affects melatonin levels following surgery^6^. This finding was also demonstrated in the study of Ram et al^10^. However, the rise in melatonin levels in the experimental group was more conspicuous.

In this study, consistent with the previous studies, the response of IL-1 secretion was not different in both control and treatment groups, whereas the increase in the IL-6 levels was greater in the treatment group^6,11^. Similar to previous studies, which showed the anti-inflammatory effects of increased levels of melatonin, we showed a significant decrease in the levels of POD 3 IL-1^2–6^. This might be related to higher levels of melatonin in the experimental group. However, the levels of POD 3 IL-6 were greater in the experimental group. We claim that this might be caused by the administration of tryptophan within parenteral nutrition. In these previous studies, melatonin was either administered or its plasma level was manipulated by sleep quality. However, in this study, plasma levels of melatonin were increased parallel to the increase in the level of tryptophan. It is well known that indoleamine 2,3-dioxygenase 1 (IDO 1) is the most essential enzyme that catalyzes the degradation of tryptophan to kynurenine^12^. IL-6 may upregulate IDO 1 expression and is closely associated with the tryptophan metabolism^13,14^. Therefore, the increased level of plasma tryptophan might have triggered an increase in IL-6 levels to induce the degradation of tryptophan in the treatment group. The CRP levels were less in the treatment group, and the difference was apparent 3 days after the surgery but statistically not different. This could be explained by the complex balance between the secretions of melatonin and IL-6. Probably, even if the difference between groups in the melatonin levels reached the highest on PODs 3 and 5, the level of IL-6 in the treatment group was greater than in the control group on POD 3 as well. Thus, probably, the net effect led to an insignificant difference in the levels of CRP.

The study has several limitations which should be reviewed. Our small sample size limited the power of the statistical analysis. Another limitation was the complexity of the relationship between tryptophan, melatonin, and immune response. Supplementation of peripheral protein, glucose, and lipids has effects on immune response and might have interfered with our results. Therefore, treatment with tryptophan after surgery would be more informative about the relationship between tryptophan, melatonin, and postoperative immune response. Furthermore, we believe that still there might be some unknown interactions. Thus, more investigations should be conducted to clarify these complex interactions.
